# Application of Neural Network Based on Visual Recognition in Color Perception Analysis of Intelligent Vehicle HMI Interactive Interface under User Experience

**DOI:** 10.1155/2022/3929110

**Published:** 2022-10-12

**Authors:** Dongxin Zhao

**Affiliations:** Huita Information Technology Consulting (Shanghai) Co.,Ltd, Shanghai 201210, China

## Abstract

As a bridge of human-computer communication, the color design of intelligent vehicle HMI interactive interface is particularly important. It is also the first guide to the driver during the driving process. The quality of its design will also directly affect the driver's senses and the driving safety of the vehicle. Therefore, this paper introduces the current situation, design principle, and future development of the vehicle interaction interface from multiple perspectives. Through the neural network system (condition generation countermeasure network model) of visual recognition, the color of the intelligent vehicle HMI interactive interface under the user experience is analyzed. According to the analysis of the psychological cognition and behavior operation of the automobile user, the correlation analysis of the human, vehicle, environment, and various elements of the interface is carried out, and how the vehicle interactive interface can meet the expected physiological and psychological needs of the user more and improve the operability is discussed in order to design an on-board HMI interactive interface that can be intelligently perceived according to weather, driver's interests, and other factors and then improve the current backward operation mode of the on-board interactive interface, so that the interaction between people and vehicles is more smooth and pleasant.

## 1. Introduction

Color perception is an important function of the human visual system, and it is also one of the most important ways for the human nervous system to receive external information. The research entity formed by separating the part responsible for color perception in the human visual system is called the color perception system.

The early research and attention on the human color vision system can be traced back to the relevant literature on the discovery of color blindness by Dalton, a famous British scientist, at the end of the 18^th^ century [1–3], which first revealed and described the specific subtle differences between the color vision system of a small number of human individuals and normal people, thus initially forming the concept of normal color vision and abnormal color vision, attracting the attention of academia to the differences between color vision systems [4, 5].

We live in a world full of color. Anything needs color decoration. Color can directly or indirectly affect people's emotions [6–10]. In the long-term life of human beings, the application of color makes it a visual symbol system, and it has certain symbolism and stability. Color is a physical phenomenon, which should have no emotional expression, but in people's long-term application, many experiences and memories are formed in people's minds. When this intuitive perception and memory collide with external color stimulation in the heart, it gives color the corresponding emotion [2, 11, 12]. Color itself is expressionless, but in the long-term life practice and experience, human beings give color unique expression characteristics. As the color changes, the color expression will change accordingly. People use some typical color expressions to create things that meet people's needs [13–16].

With the rapid development of the computer system, the automobile has become more and more intelligent. From the original simple gasoline engine, it has developed into a new product combining science and technology with wisdom. With the diversification of functions, complicated operation modes have been added. More and more car factories and emerging companies have paid attention to the informatization, interconnection, and intelligence of cars. It is no longer a concept but reflected in products and services. As an information-based product, on-board equipment has been widely used by users in daily life [17–19]. Interaction design in the car environment is very different from mobile phones and computers. The time that eyes can stay on the screen is short, and the complex environment during driving requires designers to constantly study and deepen in the process of interface design [20–22]. However, at present, most automotive systems and on-board products in the domestic market cannot meet the needs of today's users, making many seemingly powerful on-board devices difficult to operate and understand in the actual use of users. The unreasonable software interface will cause cognitive burden and operation difficulties to the driving driver when used, thus posing a certain threat to the driving safety of the driver [23, 24]. Through the research on the information visualization design of the on-board interactive interface, the usability level of the on-board interactive interface can be improved, and the interface operation of the on-board product is simpler and more effective. At the same time, it also has a certain guiding significance for the design and research of the software interface [25]. The design of automobile on-board interactive interface is relatively novel in the field of interaction. It is of certain research value to locate the interactive scenario in the automobile and deeply explore the interaction process between the driver and the on-board interface [26].

Nowadays, cars have become indispensable in people's daily life. With the rapid development of science, cars are also gradually moving towards information and intelligence, and the human-computer interaction mode has also been fully used in the automotive industry. In order to improve the safety and comfort of drivers when driving, automobile manufacturers have improved and optimized the design of on-board systems, making them integrate navigation, entertainment, and other functions. However, at present, most on-board systems only meet the functional requirements and lack attention to availability and ease of use.

Therefore, based on the elaboration of interaction design and user experience, this paper analyzes the design principles of vehicle interaction interface and uses the neural network of visual recognition to explore the design method of vehicle interaction interface that can combine user characteristics and meet user needs, so as to improve user experience.

## 2. Research on the Theory of Conditional Generation Countermeasure Network and Vehicle Interaction

### 2.1. Conditional Generation Countermeasure Network

Generating confrontation network is an unsupervised learning method of deep learning, which was first proposed by Goodfellow et al. According to its design idea based on game theory, it is composed of two parts, one is called a generator and the other is called a discriminator. The generator is used to generate false data, and the discriminator determines whether the source of the data is true or false. In the original generation countermeasure network proposed by Goodfellow et al., the input of the generator is a one-dimensional random noise vector, which is finally output as an image through a multilayer fully connected neural network.

The generation countermeasure network is widely used in image generation tasks because it can synthesize images with higher accuracy and quality and has made breakthroughs in image generation, image style conversion, and image segmentation [27, 28]. The generation countermeasure network consists of two parts, one is the generator (*g*), the other is the discriminator (*d*). The generation network *G* and the discrimination network *D* iterate with each other until the generation network *G* and the discrimination network *D* are in balance with each other.

Generate a confrontation network and define the following formula to update the weight as a learning rule:

(1) $$\begin{array}{c} L_{GAN}\left(G,D\right)=\underset{G}{\text{min}}\underset{D}{\text{max}}\left[E_{x∼P_{data}\left(x\right)}\log\left(D\left(x\right)\right)+E_{z∼P_{data}\left(z\right)}\log\left(1-D\left(G\left(z\right)\right)\right)\right], \end{array}$$

where min_*G*_ is the minimum value of the generated network error; max_*D*_ is the maximum value of network error; *z* ~ *P*_*data*_(*z*)is the real sample distribution; *x* ~ *P*_*data*_(*x*) is the random noise sample distribution; and *E* is the calculated probability mean.

In the existing generation countermeasure network, the generation network *G* collects noise *Z* as input in the hidden space. When it passes through the full connection layer, it will generate images. The discrimination network *D* is used to judge the real images and pseudo images, but there is no strict correspondence between the input and output [28, 29]. Therefore, the conditional generation countermeasure network updates the weight of the existing generation countermeasure network through the following learning rules:

(2) $$\begin{array}{c} L_{cGAN}\left(G,D\right)=\underset{G}{\text{min}}\underset{D}{\text{max}}\left[E_{x∼P_{data}\left(x\right)}\log\left(D\left(y,x\right)\right)+E_{z∼P_{data}\left(z\right)}\log\left(1-D\left(G\left(z,y\right)\right)\right)\right], \end{array}$$

where *y* is the label information corresponding to the real sample data. Adding *y* label to Gaussian noise *z* can make the result of generating countermeasure network controllable.

Pix2pix replaces noise and condition information with the mask image on the basis of *cGAN* to realize image to image conversion. In order to ensure that the similarity between input and output images is large, after generating the countermeasure loss, the countermeasure network error is generated through the regular term error control [28].

(3) $$\begin{array}{c} G^{\prime }=\text{arg}\underset{G}{\text{min}}\underset{D}{\text{max}}L_{cGAN}\left(G,D\right)+\lambda L_{L1}, \end{array}$$

where *G*′ is the total loss function of Pix2Pix.

The conditional generation countermeasure network is a variant of the original generation countermeasure network architecture. Its main feature is that the condition information is added to the input of the generator so that the generator generates data according to the conditions. In the discriminator, it can also be divided into conditional discrimination and nonconditional discrimination. The conditional discrimination is to fuse the generated data of the conditional information horse and then input it to the discriminator for correlation discrimination. The nonconditional discrimination is to input the generated data to the discriminator separately, and the discriminator makes a separate judgment on it. In the process of model construction and training, the network can generate the generated data consistent with the condition information by designing a reasonable loss function for learning.

### 2.2. Vehicle Interaction Design Theory

#### 2.2.1. Characteristics of Information Visualization in Vehicle Interactive Interface

**Figure 1:**
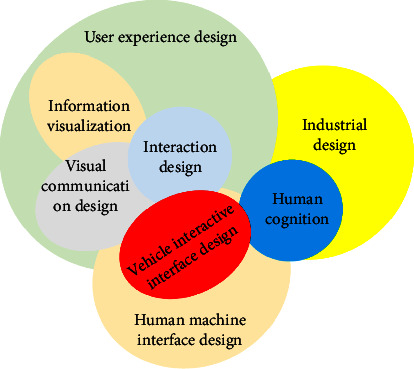
The relationship between vehicle interactive interface design and various disciplines.

The basic way for people to communicate in life is words and graphics. Since birth, people have used senses, imagination, and emotions and just have some form of dialogue with the surrounding products and environment. The design of vehicle interaction interface is a part of human-computer interaction design, and its relationship with various design disciplines is shown in Figure 1, forming an overlapping relationship with most mathematics disciplines. Information visualization design and interaction design are closely linked. Information visualization design solves the problem of information expression. On this basis, vehicle interaction design provides users with a better way to interact with information.

**Figure 2:**
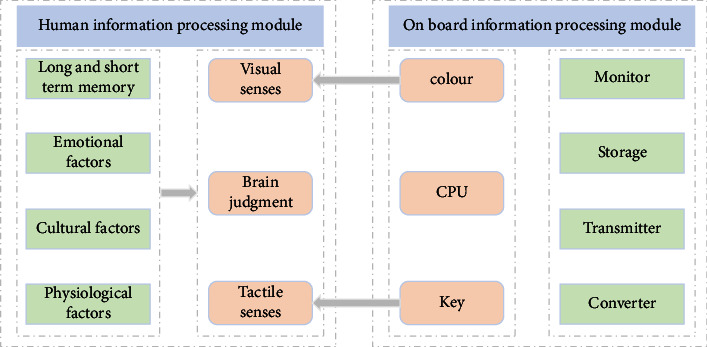
Information transmission process in vehicle interactive interface.

James Jerome Gibson, an American perceptual psychologist, believes that all the characteristics of objects can be intuitively perceived, and their information is directly expressed in vision. For example, people judge how to open a door according to the shape of the door handle. An object has more than one purpose. People will arrange their behavior according to the characteristics of the object itself. In the vehicle interactive interface, information visualization not only needs to follow the principle of information visualization design visually but also conforms to the object entity, environment, and information in the vehicle in terms of information architecture. The design of vehicle interaction interface is based on functional interface, with environmental interface as the premise and emotional interface as the center. It is clear, concise, familiar, easy to respond, easy to operate, efficient, and fault-tolerant. In the process of information transmission in the design of vehicle interactive interface, people form behavior through brain judgment and transmit it to the vehicle machine. The vehicle machine processes the input information through CPU and outputs information through color, sound, animation, shape, text, image, and so on in the interactive interface. The general information transmission process of on-board interactive interface is shown in Figure 2.

#### 2.2.2. Definition of On-Board Interactive Interface

The original car was controlled by the tiller, but in 1894, Alfred Vacheron introduced a gear system between the driver and the wheels and installed a steering wheel, which is considered to be one of the earliest human-computer interaction control devices. With the progress of time, speed indicators and fuel indicators have also been used in automobiles, becoming the earliest human-computer interactive display devices. Nowadays, all kinds of information and equipment are pouring into the vehicle, and the human-computer interaction in the vehicle becomes more and more troublesome. However, because the iteration speed of the software system is far faster than that of *R*&*D* of the automobile enterprises, the human-computer interaction development in the automobile is not as fast and perfect as the mobile phone interaction. Since the automobile has been well known by the public, the automobile, as a means of transportation for people, has not changed much in essence, but users' expectations for the automobile will be higher and higher, and car companies tend to be more functional and intelligent when designing cars. Panoramic sunroof, fingerprint identification, head up display, heated seats, and other new technologies are used in cars. All kinds of operations in the whole process of using the car from the user belong to the category of car interaction, and the design of on-board interactive interface pays more attention to the interactive interface. The interface is the contact level in the process of interaction between the human and machine. The interactive interface is expressed through sensory experience, emotional experience, and cultural experience. The sense of security and control obtained by the user through visual hearing reflects the user's concept, consciousness, lifestyle, and other cultural connotations. The user carries out an operation, and the operation result or state is feedbacked by the vehicle machine. After the user perceives the change of information through vision, hearing, touch, etc., he analyzes and explains the meaning of information through thinking and finally makes a decision. The on-board interactive interface is the link between people and vehicles. Good interface design can make communication more accurate and pleasant.

Vehicle interface can be roughly divided into three types: hard interface, soft interface, and multichannel interface:

1. Hardware interaction refers to the user's active operation behavior through the physical controller, such as using the knob to adjust the volume, using the door handle to open the door, and so on. It includes traditional controllers and parts of product entities that can be operated by users. This kind of interactive interface is more accurate in operation, and the button layout position is relatively fixed, which makes it easier to form habitual operation actions. However, the feedback is relatively low, and the feedback after the operation is not clear enough.
2. Software interaction interface refers to the behavior of presenting information through the electronic screen, and users interpret information or operate, such as setting the navigation destination through the on-board central control screen, and users observe the sudden speed information in the instrument panel, etc. There are touch operation interfaces and information only interfaces in the software interaction interface. Generally, high-resolution touch display devices are used to carry information and realize operation. This kind of interactive interface is more diverse in form, and it is not restricted by the shape and size compared with the hardware interactive interface. However, the accuracy of operation is relatively low, and the operation feedback is good. It is more in line with the user habits under the rapid development of mobile interaction.
3. Multichannel interaction refers to the process of interacting with the car through multiple sensory organs in the car environment, such as voice control, mobile phone interconnection, somatosensory control, line of sight control, and so on. It is not only through the button generator screen to interact with the car. In the car environment, when the user's hands and eyes are in use, they can use multichannel senses to interact with the car, make full use of human multisensory channels, and enrich the possibility of human car interaction at a deeper level.

### 2.3. Classification of Color like Perception

Human perception of color vision has individual differences. Generally speaking, human color vision can be divided into normal color vision and abnormal color vision. The color perception system is mainly responsible for the perception of color by the special photoreceptor on the retina, namely the cone cells. Different types of cone cells have different absorption of visible light. The superposition value of the response results of cone cells stimulated by various bands of light in the visible spectrum determines the color form of the spectrum in human eyes. In the human color vision system, there are mainly three kinds of cone cells, namely *L*-cone cells, *M*-cone cells, and *S*-cone cells [9]. For the normal color vision system, L-cone cells are most sensitive to the long wavelength of 535 nm–575 nm in the visible spectrum, M-cone cells are most sensitive to the medium wavelength of 500–550 nm in the visible spectrum, and S-cone cells are most sensitive to the short wavelength of 400–450 nm in the visible spectrum. When these three cones are absent or their sensitivity to their respective wavelength bands is shifted, the perception of color by the color vision system will change, resulting in abnormal color vision. According to the deviation degree of *L*, *M*, and *S*-cone cells in the abnormal color vision system after stimulation of different wavelength components in the spectrum or due to their own lacking, abnormal color vision can be divided into the following categories:

1. Monochromatic color vision: It is also called total color blindness. This color vision system lacks all visual cones or only one type of visual cones. According to the three primary color theory, its perception of different colors cannot be superimposed like normal color vision. It can only perceive the changes in the brightness of natural light and can only distinguish the brightness changes from black to gray and then to white but cannot or can hardly perceive other forms of color.
2. Dichromatic color vision: It is also a typr of color blindness. This color vision system lacks a type of cone cells, so it cannot effectively perceive the spectral information of the corresponding band, and there is a perceptual deviation. According to the type of visual cone cells missing, those missing L-cone cells are generally called red blindness, which is mainly manifested in the inability to normally perceive the red band spectrum. The absence of *M*-cone cells is called green blindness, which is mainly manifested in the inability to normally perceive the green band spectrum. Those who lack *S*-cone cells are called to suffer from blue blindness, and their main performance is that they cannot normally perceive the blue purple spectrum. In abnormal color vision groups, the first two kinds of deficiencies are in the majority, which are often collectively referred to as red and green color blindness; The number of blue blindness caused by the third condition is very small.
3. Trichromatic color vision: It is color weakness. In this type, cone cells is almost the same as the normal color vision system, but one type of cone cells has some defects or abnormalities, and the perception of the spectrum of the corresponding band has changed, resulting in confusion and deviation in color resolution, and dichromatic color vision types can be subdivided into red weak, green weak, and blue weak according to their defective cone cell types, and they can be classified according to their severity. From the perspective of color perception, when the color weakness is slight to the extreme, its condition is close to normal color perception. When it reaches the most serious level, its condition is close to color blindness.

## 3. Structure Design of Generative Antagonism Neural Network Based on Visual Recognition

**Figure 3:**
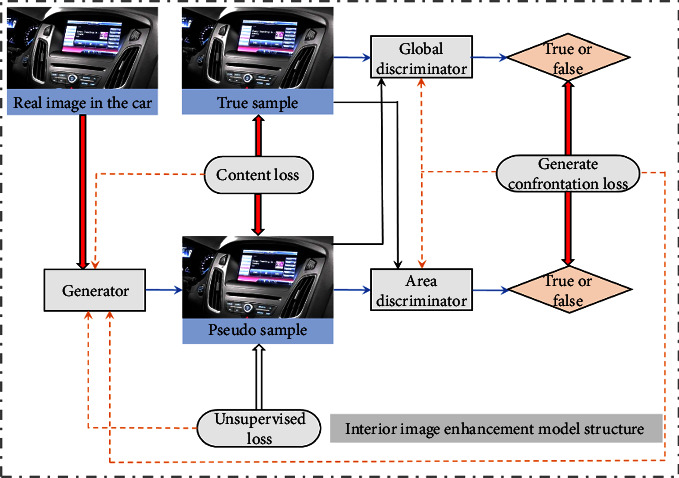
Interior image enhancement model structure.

In order to solve the problem of color perception in the vehicle HMI interactive interface, this paper proposes an image enhancement model based on conditional generation countermeasure network, as shown in Figure 3. It is mainly composed of two parts, generation network and discrimination network. The former is used to learn the data distribution of clear images and obtain the nonlinear transformation relationship between the vehicle color image and this distribution. Then generate a pseudo clear image that removes the influence of other factors.

**Figure 4:**
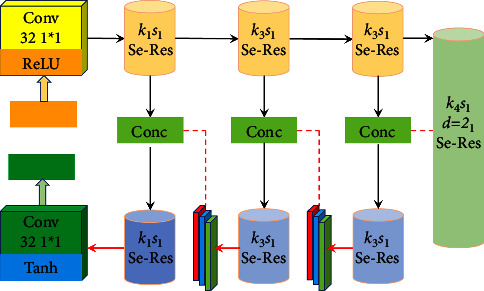
Design of generation countermeasure network structure.

The generated network structure is shown in Figure 4, where *k*_*i*_ is *i* × *i* core size, *s*_*j*_ is the moving stride with length *j*, *D* is the expansion rate in cavity convolution, Conv is convolution operation, and Conc is a splicing operation. The latter discriminates the authenticity of the input image, the clear reference image label is true, and the pseudo clear image label obtained from the generated network is false. In addition, the discriminant network adopts the double discriminant form, which can supervise the global style and detail edges, respectively. In the loss function, this paper designs an unsupervised form that only considers the quality of the image itself, which together with confrontation and content loss constrains the training direction of the model and helps to alleviate the dependence on data. The network design will be described in detail in Figure 4.

### 3.1. Generate Network Structure Design

The network input is 256 × 256 × 3 pixels of image data through 32 channels 1 × 1 convolution operation, and the ReLU activation function gets the characteristic distribution of 256 × 256 × 32. First, in order to avoid the gradient sparsity problem, this model uses basic convolution operation to replace the pooling layer in the original se res module, so as to prevent information loss and help improve the stability of the network. At the same time, in order to realize the fusion of multiscale feature information, this paper, based on the original se res module and different sizes of convolution kernels are selected according to the convolution position. In addition, the kernel size of convolution operation will affect the parameters and calculation of the network. Therefore, in this paper, 3 × 3 hole convolution is used to replace 7 × 7 traditional convolution in the dark green module, which not only ensures the receptive field but also reduces the complexity of the network [30]. *D* is set to 2. After four se res module operations, convolution processing with the same size is used in the symmetrical position. At the same time, in order to improve the utilization of information and prevent feature loss, this paper adds multiple direct channels and uses the splicing operation to ensure the complete transmission of low-level features. After splicing, the feature distribution is 64 channels, which are used as the input of the last three se res modules. In addition, before the splicing operation, add three-layer networks, including yellow, gray, and blue ones which are convolution operation, nonlinear activation, and normalization, respectively. The convolution selection of the output layer is 4 × 4. The activation function is set as tanh, and the tanh activation output can play the role of both activation and normalization. It normalizes the calculation results between [−1, 1], which can avoid too large or too small values. During training, all image data are normalized to [−1, 1] and then inversely adjusted to [0, 255] when the generated results are displayed, so the value of Tanh activation function is adjusted as the pixel value of the three channels of the output image, Thus, the final enhancement result can be obtained.

### 3.2. Discriminant Network Structure Design

**Figure 5:**
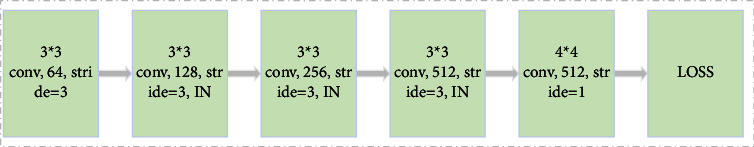
Discrimination network structure design diagram.

In the process of network training, the ability of generating network and distinguishing network cannot always maintain a balance, resulting in the inability of generating network to distinguish network to compete, and limiting the network depth of distinguishing network limits the ability of distinguishing network to a certain extent. The discriminant network structure is shown in Figure 5, which is composed of five convolution layers without using full connection and pooling layers [31, 32].

According to Figure 5, since the batch normal layer may bring artifacts to the generated pictures during network training, and the network training performance cannot be stabilized, it is judged that the batch normal layer is not used in the network, and it is replaced by the instance normal layer. Using the instance normal layer helps to enhance the network performance and reduce the computational complexity. In addition, the instance normal layer can subtract the depth information in the network input direction from the mean divided by the standard deviation, accelerate the network training speed, and increase the nonlinear fitting ability. Therefore, it is replaced by the instance normal layer.

Optimize the activation function of the last layer. The activation function in CNN network generally adopts the Sigmoid function or ReLU function. When the network training ability is strong, it can 100% distinguish the false image of the generated network. RelU, which is often used as the activation function in deep learning, can speed up the calculation of convolution. However, when a very large gradient flows through ReLU neurons, it may disturb the distribution of data, resulting in some neurons not activating any data and turning off. For this situation, use the leaky ReLU function, which can avoid this situation. The definition of the leaky ReLU function is as follows:

(4) $$\begin{array}{c} y=\text{max}\left(0,x\right)+leaky*\text{min}\left(0,x\right), \end{array}$$

where leaky is the coefficient of (0, 1).

**Figure 6:**
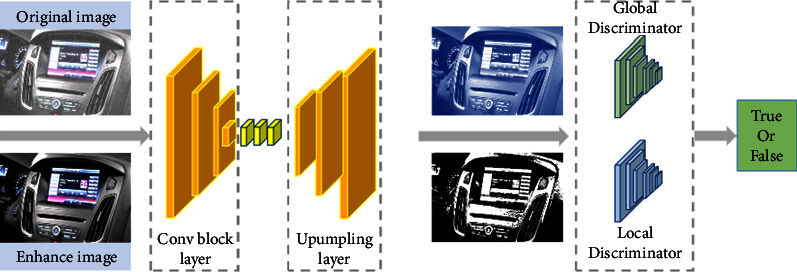
Overall frame diagram of color perception.

To sum up, the overall framework of realizing the color perception of the on-board HMI interactive interface through the condition generation countermeasure network is shown in Figure 6.

## 4. Color Perception Experiment and Result Analysis of Vehicle HMI Interface Based on Generative Antagonism Neural Network

### 4.1. Experimental Preparation

In order to get the user's favorite car interface color design scheme in a more objective way, the color perception experiment of car instrument and central control interface based on eye movement physiological data is designed. The experiment needs to be carried out under quiet conditions, and the electronic equipment irrelevant to the experiment in the same space should be closed in the laboratory. The experiment should be carried out in an environment with good light, smooth ventilation, and comfortable room temperature, and the subjects should be in a happy state to avoid the impact of measurement on the experimental results due to tension, anxiety, or depression.

This experiment was improved and arranged on the basis of existing research. There were 20 subjects in the test, of which the male to female ratio was 1 : 1. The age range of the subjects is 20–27 years old. Among them, the ratio of researchers engaged in automobile styling design to nonprofessionals is 1 : 1. This test requires that the test personnel participating in the test shall not engage in any violent activities, drink alcohol, keep regular work and rest, and shall not smoke, drink strong tea, drink coffee, or take drugs related to the nervous system before the test.

**Figure 7:**
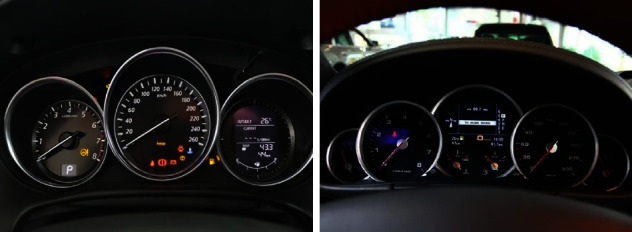
Legend of the experimental sample.

A total of 120 images of instruments and central control of 60 different models are classified, and these images are optimized for image processing such as de reflection and size correction. By consulting official pictures and video materials, we can grasp the color design style of each picture and carry out appropriate color correction, so as to ensure the true color of the picture as much as possible and reduce the color difference. Each sample picture includes six models, so 20 samples of instruments and central control are produced, including 10 samples of instruments and 10 samples of central control. The background of the experimental sample is pure black, and the size of each sample is 973 mm × 598 mm.  Figure 7 is an example of an experimental sample picture.

### 4.2. Experimental Scheme

In the preparation stage of the experiment, it is necessary to check and debug the experimental equipment, confirm that the storage space of the power and data of the equipment is enough to meet the needs of this experiment, and prepare the corresponding emergency plan. The detailed experimental steps are as follows:

  Step 1: Adjust the posture of the subjects to ensure that the sitting posture of all subjects is correct
  Step 2: The subjects are required to sit at the designated position and adopt a normal and comfortable sitting posture, with their backs on the backrest and their eyes parallel to the center of the screen
  Step 3: Present the pictures prepared in advance to the subjects, and let the subjects record the pictures they have selected and describe the colors that have been washed and matched;
  Step 4: Use all the pictures selected in the test to generate the countermeasure network for learning analysis and extract the features of the analyzed pictures
  Step 5: Count the most popular group matching colors
  Step 6: Summarize the experimental records.

### 4.3. Evaluation Index Design

The experimental evaluation of the two proposed methods of color vision test map synthesis is mainly carried out from two perspectives. First, evaluate the quality of a single synthetic image. Using the two color vision test chart synthesis methods proposed in this paper, to generate a group of images, randomly select one from the images for subjective and objective evaluation and illustrate the effectiveness of the method proposed in this paper and its advantages compared with the traditional test chart picture book through numerical comparison with the classic images in the color vision test chart picture book in terms of compliance and specificity.

### 4.4. Analysis of Experimental Results

**Figure 8:**
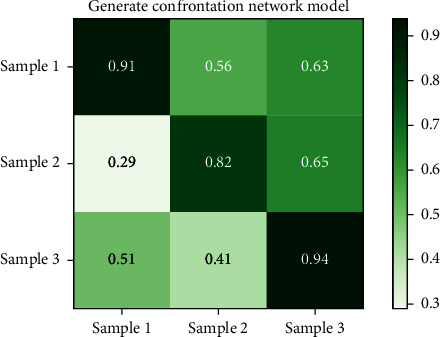
Generate compliance results for the confrontation model.

**Figure 9:**
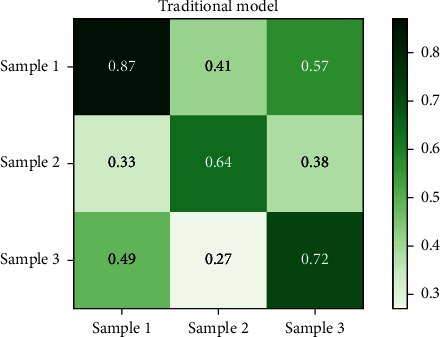
Legend of the experimental sample.

Figures 8 and 9, respectively, show the compliance results of the on-board HMI interactive interface color perception model based on the antineural network model generated in this paper and the traditional color perception model for the above color perception test chart.  Figure 8 shows the compliance results of the model constructed in this paper, and Figure 9 shows the compliance results of the traditional color perception model. From the analysis results of the compliance of the models in Figures 8 and 9 for color perception, it can be seen that the generation antagonism neural network model constructed in this paper has better compliance for color, and its maximum compliance is close to 0.94, while the traditional model is only 0.87.

**Figure 10:**
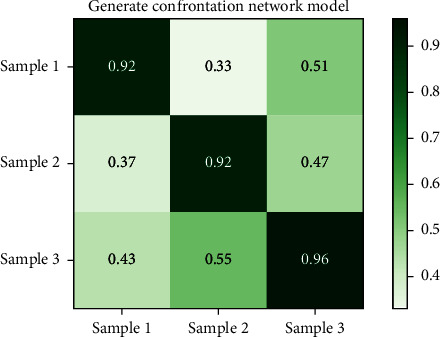
Generate specific results of the confrontation model.

**Figure 11:**
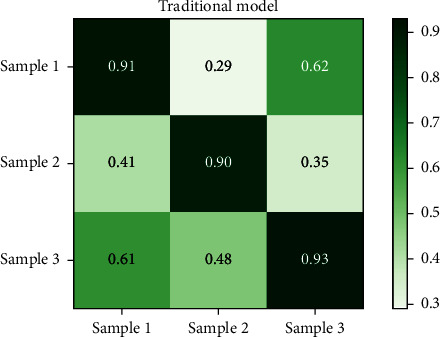
Specific results of traditional models.

Figures 10 and 11, respectively, show the specificity results of the vehicle HMI interactive interface color perception model based on the antineural network model generated in this paper and the traditional color perception model for the above color perception test chart, of which Figure 10 is the specificity results of the model constructed in this paper and Figure 11 is the specificity results of the traditional color perception model. It can be seen from the specificity analysis results of the models in Figures 10 and 11 that the specificity of the generation antagonism neural network model constructed in this paper for color is basically equivalent to that of the traditional model, and the maximum specificity of both models is above 0.9.

Based on the above compliance and specificity results, it is found that, first of all, from the compliance of Figures 8 and 9, the compliance of the two different models for color matching has a large deviation, in which the opportunity generation confrontation neural network model constructed in this paper is significantly better than the traditional model. In terms of specificity, it can be seen from Figures 10 and 11 that the specificity of the two models is similar. It can be seen that the generative antagonism neural network model based on this paper not only retains the color perception ability of the original model but also can match the colors well, which verifies the applicability of the model constructed in this paper.

## 5. Conclusion

There are many colors on the vehicle mounted HMI interactive interface. However, among the many colors on the vehicle mounted interactive interface, many colors do not conform to the human body design, or the user experience is insufficient, which may easily lead to the reduction of driving pleasure and even increase the probability of causing traffic accidents. In view of the above problems, this paper analyzes the color of the intelligent vehicle HMI interactive interface under the user experience through the neural network system of visual recognition (condition generation confrontation network model), analyzes the correlation of various elements of human, vehicle, environment, and interface according to the analysis of the psychological cognition and behavioral operation of vehicle users, and discusses how the vehicle interactive interface can better meet the expected physiological and psychological needs of users and improve the operability, so as to design an on-board HMI interactive interface that can be intelligently perceived according to factors such as weather and driver's interest so as to improve the current backward status of the operation mode of the on-board interactive interface. On the basis of elaborating the interactive design and user experience, by analyzing the design principles of the on-board interactive interface, the neural network of visual recognition is used to explore on how to combine the characteristics of users. The design method of vehicle interaction interface meets the needs of users so as to improve the user's sense of experience.

## Data Availability

The dataset used in this paper are available from the corresponding author upon request.
